# The Role of Endophytic Fungal Individuals and Communities in the Decomposition of *Pinus massoniana* Needle Litter

**DOI:** 10.1371/journal.pone.0105911

**Published:** 2014-08-26

**Authors:** Zhilin Yuan, Lianqing Chen

**Affiliations:** Institute of Subtropical Forestry, Chinese Academy of Forestry, Fuyang, Zhejiang Province, P. R. China; Helmholtz Centre for Environmental Research (UFZ), Germany

## Abstract

The role of fungal endophytes (FEs) as “pioneer” decomposers has recently been recognized; however, the extent to which FEs contribute to litter loss is less well understood. The genetic and enzymatic bases of FE-mediated decomposition have also rarely been addressed. The effects of populations and individuals (with an emphasis on two dominant *Lophodermium* taxa) of FEs on needle-litter decomposition were assessed for *Pinus massoniana*, a ubiquitous pine in southern China. Data from *in vivo* (microcosm) experiments indicated that the percentage of litter-mass loss triggered by FEs was linearly correlated with incubation time and approached 60% after seven months. *In vitro* decomposition tests also confirmed that endophytic *Lophodermium* isolates caused 14–22% mass loss within two months. Qualitative analysis of exoenzymes (cellulase and laccase, important for lignocellulose degradation) revealed that almost all of the *Lophodermium* isolates showed moderate or strong positive reactions. Furthermore, partial sequences of β-glucosidase (glycoside hydrolase family 3, GH3), laccase, and cellobiohydrolase (GH7) genes were amplified from *Lophodermium* isolates as “functional markers” to evaluate their potential for lignocellulolytic activity. Three different genes were detected, suggesting a flexible and delicate decomposition system rich in FEs. Our work highlights the possibility that the saprophytism and endophytism of FEs may be prerequisites to initiating rapid decomposition and thus may be key in Fes’ contribution to litter decomposition, at least in the early stage. Potential indicators of the presence of core fungal decomposers are also briefly discussed.

## Introduction

Microbes (especially saprotrophic fungi) are the major drivers of litter decomposition due to their striking species diversity and degradation capacity [1]. Decomposition is accompanied by dynamic and rapid succession in the litter-associated microbial community, as indicated by DNA- and RNA-based detection assays [2]–[5].

There is a large body of previous work on the soil and litter-layer fungi involved in decomposition. Basidiomycetous fungi are considered more effective decomposers than ascomycetes because the former typically have genes that encode for laccase and cellobiohydrolase [6]–[7]. Recent studies, however, indicate that ascomycetes have similar or equal performance in litter decomposition [8]. Furthermore, clear evidence now indicates that plant fungal endophytes (FEs) act as “pioneer” decomposers because of their persistence in live, senescent or dead inner plant tissues [2], [9], [10]. When plant organs undergo senescence, FEs switch from obligate endophytism to facultative saprophytism [11]–[15].

Taxonomically, most endophytes (or the dominant species) are ascomycetes, suggesting that FE-mediated litter decomposition is far more important than previously thought [16]. In addition, multiple exoenzymes produced by endophytes have been extensively studied [17]–[18]; however, the extent to which FEs contribute to litter loss is less well understood. The genetic and enzymatic bases of FE-mediated decomposition have also rarely been addressed.

Masson pine (*Pinus massoniana* L.) is a ubiquitous species in southern China and accounts for more than 50% of the total subtropical forest area, suggesting its key roles in nutrient and carbon cycling in forest ecosystems. Most current work focuses on how litter decomposition responds to environmental changes such as temperature and nitrogen (N) deposition [19]–[20]. To our knowledge, no previous study has examined the role of microbes in *P. massoniana* litter decomposition. Here, we examine (1) whether endophytic fungal populations and individuals cause mass loss, (2) the extent of decomposition caused by these fungi and (3) whether fungal endophytes contain a rich exoenzyme-secreting system, which has been widely recognized as an important marker of ligninolytic microbes.

## Materials and Methods

### Isolation and molecular identification of dominant FEs

For the *in vitro* decomposition test, we first isolated FEs from mature, healthy needle tissues. The State Forestry Administration of P. R. China issued a permit for sample collection in this area. The sampling site was located at Laoshan Forest Farm, Zhejiang Province (N 29°32′34′′, E 119°04′04′′). Monoculture plantations of 24-year-old *P. massoniana* were chosen for study. Intact twigs bearing conifers were removed from seven trees and taken back to the laboratory within 12 h. The surface-sterilization procedure was conducted according to Yuan *et al*. [21]. Fragmented needles 0.5 cm in length were placed on 2% malt-extract agar (MEA). After incubation for one week at 20°C, the hyphae emerging from both ends were cut and then transferred onto potato-dextrose agar for purification. All obtained isolates were grouped into several morphotypes based on the criteria defined by Lacap *et al.* [22], including colony appearance, growth rate, and color. Although previous work has compared the decomposition ability of rare and common FEs [23], we focused on dominant species because (1) rare fungi showed very slow growth, indicating weak or no decomposition; (2) dominant fungi always occupied a large number of spatial niches, indicating their active involvement in litter decomposition; (3) studies of conifer decomposition have described the importance of dominant needle endophytes (*Lophodermium piceae* and *Lophodermium pinastri*) as primary decomposers [24]–[25]. In the present study, two morphotypes (1 and 2) were considered dominant colonizers because of their high isolation frequencies of 55.2% and 32%.

As the dominant fungi do not form sporulating structures on artificial media, a molecular identification approach was used. The internal transcribed spacer (ITS) rDNA region was amplified using the universal fungal primer pair ITS1F and ITS4 [26]–[27]. In total, 34 isolates of dominant fungi were selected for sequencing. BLAST searches of ITS sequences against the National Center of Biotechnology Information (NCBI) database returned close matches for these fungi. All sequences were aligned in MEGA (version 5.2) using Muscle algorithms. Ambiguously aligned regions were excluded from the phylogenetic analyses. The alignment was then manually modified using GENEDOC [28] when necessary. All sequences were deposited in GenBank, and the accession numbers are listed in Table 1. A maximum-likelihood tree was constructed using the RAxML BlackBox (http://embnet.vital-it.ch/raxml-bb/) with the gamma-partitioned model. Node support was assessed with 100 rapid bootstrap replicates.

**Table 1:** The PCR reaction conditions and components for amplification of ITS1-5.8S-ITS2, cellobiohydrolase, β-glucosidase, laccase genes in endophytic *Lophodermium* isolates.

	Genes for amplification			
	ITS	cellobiohydrolase(GH7 family)	β-glucosidase(GH3 family)	laccase
PCR reaction mixture(50 µl)	25 µl 2×Taq MasterMix buffer (containing PCR buffer, dNTPs, Mg^2+^, and Taq DNA Polymerase),0.5 µl of each primer (50 µM)3 µl template (10–50 ng)21 µl RNase-free H_2_O			
PCR conditions	4 min at 94°C35 cycle40 s at 94°C50 s at 55°C1 min at 72°C10 min at 72°C	4 min at 95°C35 cycle40 s at 95°C1 min at 48°C1.5 min at 72°C10 min at 72°C	4 min at 94°C35 cycle50 s at 94°C50 s at 50°C1.5 min at 72°C10 min at 72°C	4 min at 95°C35 cycle50 s at 95°C1 min at 47°C3 min at 72°C10 min at 72°C
Primer sequences(5′→3′)	ITS1F: CTTGGTCATTTAGAGGAAGTAAITS4: TCCTCCGCTTATTGATATGC [27]	fungcbhIF: ACCAAYTGCTAYACIRGYAAfungcbhIR: GCYTCCCAIATRTCCATC[32]	Glc1_155F:GGIMGIAAYTGGGARGGNTTGlc1_235R:AYIGCRTCIGCRAANGGCCA[8]	LAC2FOR:GGIACIWIITGGTAYCAYWSICALAC3REV: CCRTGIWKRTGIAWIGGRTGIGG[33]
Accession number	KJ847874–KJ847907	KJ847908–KJ847940	KJ847941–KJ847968	KJ847846–KJ847873

Note: Ambiguous bases are defined as follows: M, A/C; R, A/G; W, A/T; Y, C/T; S, C/G; K, T/G; I, inosine.

### In vivo (microcosm) decomposition assay at the community level

To evaluate the effect of the natural FE community on litter decomposition, an *in vivo* (microcosm) assay was performed as described by Müller *et al*. [9] and Korkama-Rajala *et al*. [3]. Briefly, the intact needles were cut at the base of branches originating from seven trees growing in the same stand. All needles were pooled and then divided into 50 batches (4 g fresh weight per batch). Eight batches of needles were dried at 80°C for 72 h until they reached a constant weight to determine the dry weight of the fresh needles. After surface sterilization, there are 42 remaining batches were placed on the surface of 150 g of autoclaved soil collected from monoculture *P. massoniana* forests in a 500 ml flask. The flasks were closed with sterile rubber plugs and incubated at 20°C for 12 h under controlled light and 85% relative humidity to prevent drying. Six flasks were sampled per month for seven months. To determine their dry weight, the needles were rinsed in water and then dried as described above. The results are expressed as mean ± standard deviation (SD).

### *In vitro* decomposition assay at the individual level

Thirteen *Lophodermium* isolates (seven *Lophodermium* sp. 1 and six *Lophodermium* sp. 2) were evaluated in terms of their capacities to cause mass loss in *P. massoniana* needles *in vitro*. Healthy needles were divided into 70 batches and sterilized for 30 min at 121°C. Each batch was dried at 70°C to a constant mass, which was then recorded. The needles were placed on the surface of Petri dishes (12 cm diameter) containing 50 ml SNA medium (synthetic low-nutrient agar; 1 g KH2PO4, 1 g KNO3, 0.5 g MgSO4·7H2O, 0.5 g KCl, 0.2 g glucose, 0.2 g sucrose, 20 g agar, 1 l water), inoculated with five mycelia plugs adjacent to the needles. Five replicates of each fungal isolate were examined, and five un-inoculated plates served as a control. The plates were sealed with two layers of Parafilm and incubated for 2 months at 20°C in darkness. After incubation, the needles were also dried and weighed, and mass loss was determined as a percentage of the original mass.

### Qualitative assessment of laccase and cellulase production by Lophodermium isolates

The laccase and cellulase produced by *Lophodermium* isolates, which are responsible for lignocellulose degradation, were measured qualitatively according to a previously described procedure [29]. The syringaldazine well test was used to evaluate laccase activity, and dye staining in carboxymethylcellulose agar (CMC agar) was used to test for cellulase. Isolates used in the *in vitro* decomposition assay were cultured on liquid basal medium (LBM) supplemented with 1.6% w/v agar and 20% w/v glucose. After 10 days of incubation at 25°C in darkness, 50 µl 0.1% w/v syringaldazine was added to wells cut from the edge of the fungal colony. A positive reaction for laccase was indicated by the appearance of a purple color around each well. Similarly, isolates were cultured on cellulolysis basal medium (CBM) for several days under the same conditions until the colony diameter exceeded 40 mm. The plates were flooded with 2% w/v aqueous Congo red and left for at least 15 minutes. Clear zones around colonies indicated cellulase activity.

### Amplification of partial genes of β-glucosidase, laccase and cellobiohydrolase in endophytic *Lophodermium* isolates

The three partial functional genes were amplified from genomic DNA from the 34 representative *Lophodermium* isolates. The primer sets and PCR conditions are summarized in Table 1. Bands of the expected size were excised from 1.5% agarose gel (for the β-glucosidase gene, two bands close together were also excised separately). The PCR products were purified using a DNA Gel Extraction Kit (Axygen, China) and then ligated into the pUCM-T vector (Sangon, China) and transformed into *Escherichia coli* DH5α according to the manufacturers’ instructions. Cloned products were sequenced using the M13for and/or M13rev primers.

### Sequence alignment and phylogenetic analysis

The raw sequences were trimmed to remove the vector sequences using VecScreen (www.ncbi.nlm.nih.gov/tools/vecscreen/) and then subjected to a BLASTx search for sequence homology. We compared the similarity of the amino acid sequences to the closest matches to determine the positions of the putative introns. The obtained coding sequences were translated into amino acids and then aligned using Clustal X. Phylogenetic analyses were performed with the PHYLIP 3.68 package using Kimura’s method for amino acid comparisons (Program PROTDIST). Distance trees were constructed using the neighbor-joining (NJ) method. Bootstrap values were calculated using Seqboot (1000 replicates), Protdist, Neighbor, and Consense. The DNA sequences without introns and deduced amino acid sequences were deposited in GenBank. The accession numbers are shown in Table 1.

### Ethics statement

Our study does not require an ethics statement.

## Results

### Molecular identification of dominant FE groups in *P. massoIIniana* needles

**Figure 1:**
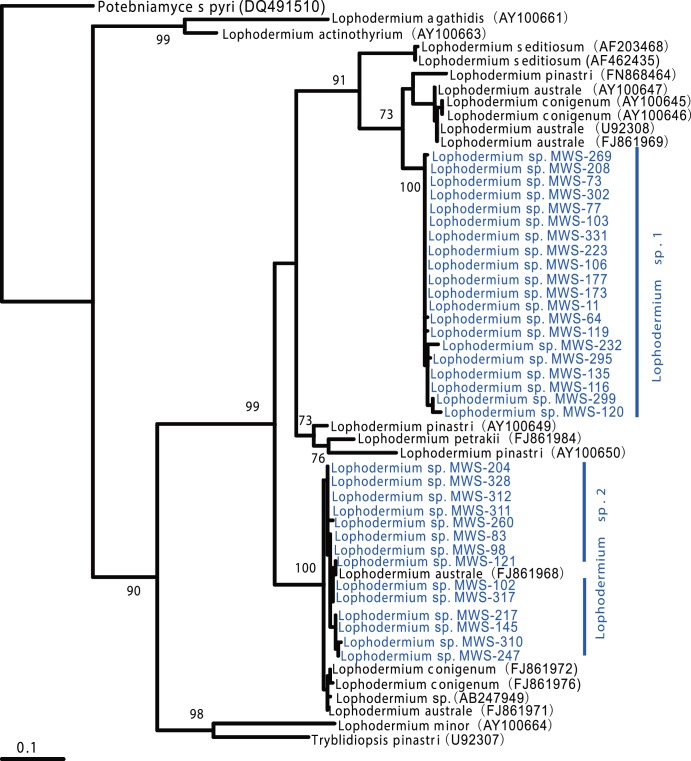
Maximum-likelihood phylogenetic analysis of ITS sequences between *Lophodermium* isolates and their close members. *Potebniamyces pyri* was used to root the tree. Branch-support values are indicated by numbers near branches.

**Figure 2:**
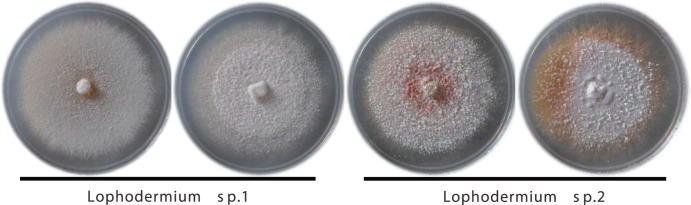
Representative *Lophodermium* isolates recovered from *P. massoniana*. All isolates were grown on PDA in the dark at 25°C for one week.

Based on the BLAST results from the ITS sequences, 34 dominant FE isolates from *P. massoniana* were assigned to the family Rhytismataceae (Ascomycota). Phylogenic analysis revealed that these two dominant morphotypes were genetically close to the *Lophodermium* genus but clearly belonged to two groups: *Lophodermium* sp. 1 and *Lophodermium* sp. 2 (Fig. 1). *Lophodermium* sp. 1 formed a distinct clade and did not cluster with any known species in this genus, representing a potentially novel taxon. *Lophodermium* sp. 2 showed a strong affinity for *L. australe* and *L. conigenum*. As *Lophodermium* sp. 2 remained sterile on artificial media, it could not be identified to the species level (Fig. 2). Our data also revealed that the morphotypes truly reflected taxonomic units, as the two morphotypes corresponded to two related taxa. These isolates were used for the subsequent experiments except for the *in vivo* decomposition assay.

### Decomposition by the natural FE population

**Figure 3:**
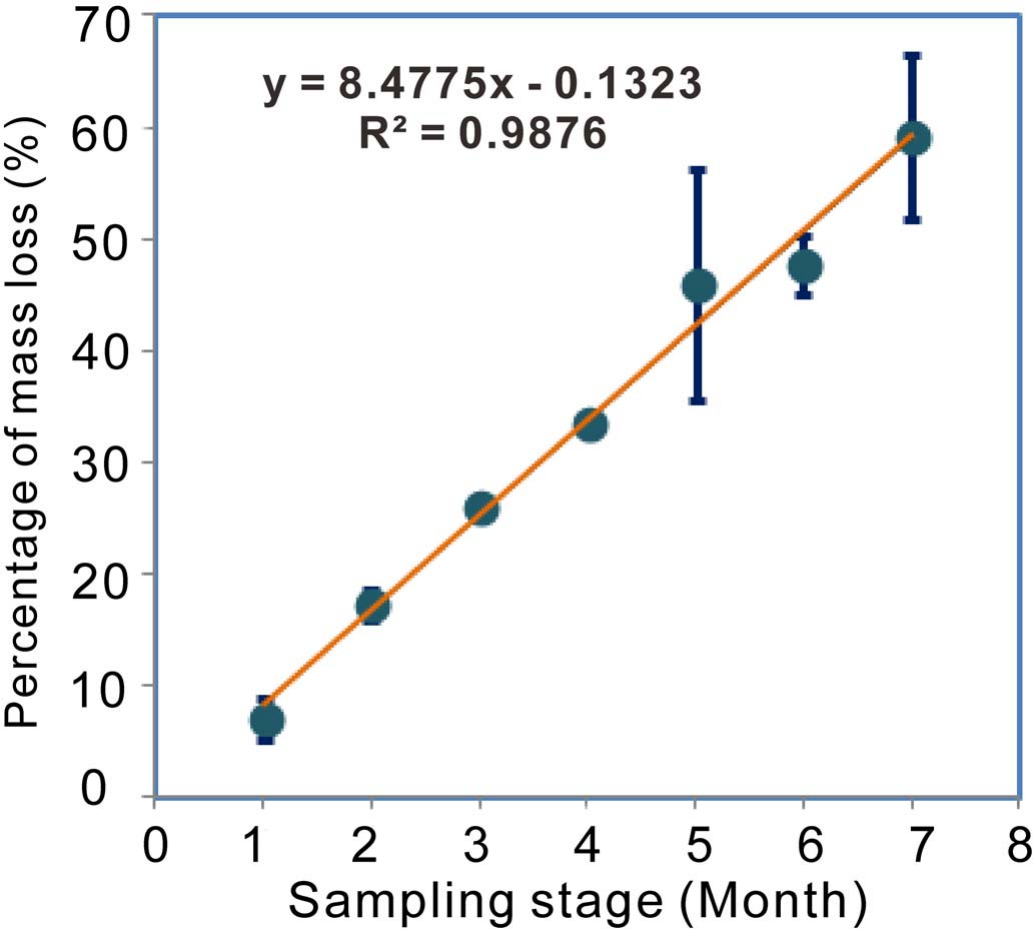
*In vivo* assay showing the relationship between percentage of mass lost and sampling stage in FE-mediated decomposition.

Using *in vivo* methods (microcosm) that simulate a natural environment, we evaluated decomposition by the natural FE population in needle litter over half a year. The relationship between percentage mass loss and litter incubation time was assessed by linear regression (Fig. 3). The data indicated that the percentage of litter mass loss increased gradually, approaching 60% after seven months of incubation. The coefficient of determination (R2 value) for this relationship was 0.9876, suggesting that the percentage of mass loss was highly correlated with incubation time. The final sampling date was set when the litter had become soft and brown, when the fungal mycelium had completely colonized the needle surface.

### Decomposition by Lophodermium isolates

**Figure 4:**
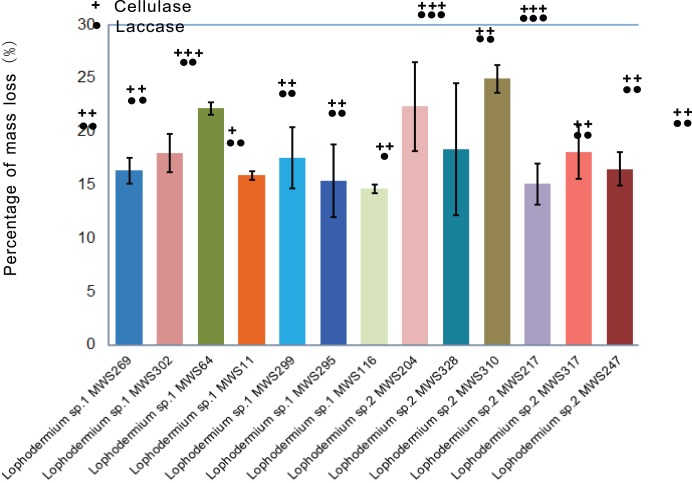
*In vitro* decomposition ability of thirteen *Lophodermium* isolates. “+”, “++” and “+++” indicate the cellulase activity from weak to strong. Similarly, “•,” “••” and “•••” indicate the laccase activity from weak to strong.

The mass loss of *P. massoniana* needles caused by pure *Lophodermium* cultures (seven isolates of *Lophodermium* sp. 1 and six isolates of *Lophodermium* sp. 2) was investigated *in vitro*. As shown in Fig. 4, all isolates resulted in 14–22% mass loss after two months of incubation. There was no clear difference between the two *Lophodermium* groups in terms of their contribution to decomposition and enzyme production. Most isolates showed moderate or strong cellulase and laccase activity.

### Detection and phylogeny of β-glucosidase, laccase and cellobiohydrolase in endophytic Lophodermium isolates

**Figure 5:**
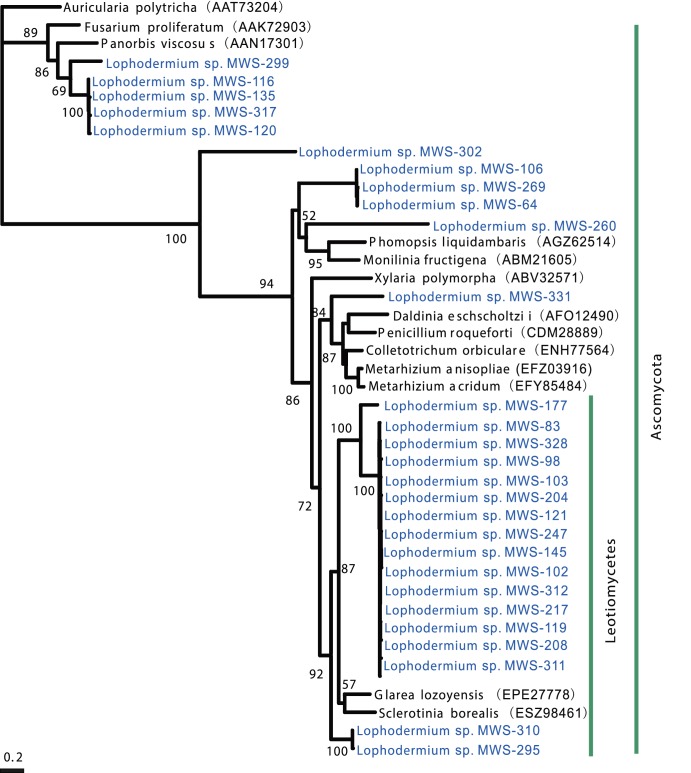
Phylogenetic tree of the partial laccase amino-acid sequences of the *Lophodermium* isolates and their close relatives. Bootstrap values greater than 50% are indicated at branch nodes.

**Figure 6:**
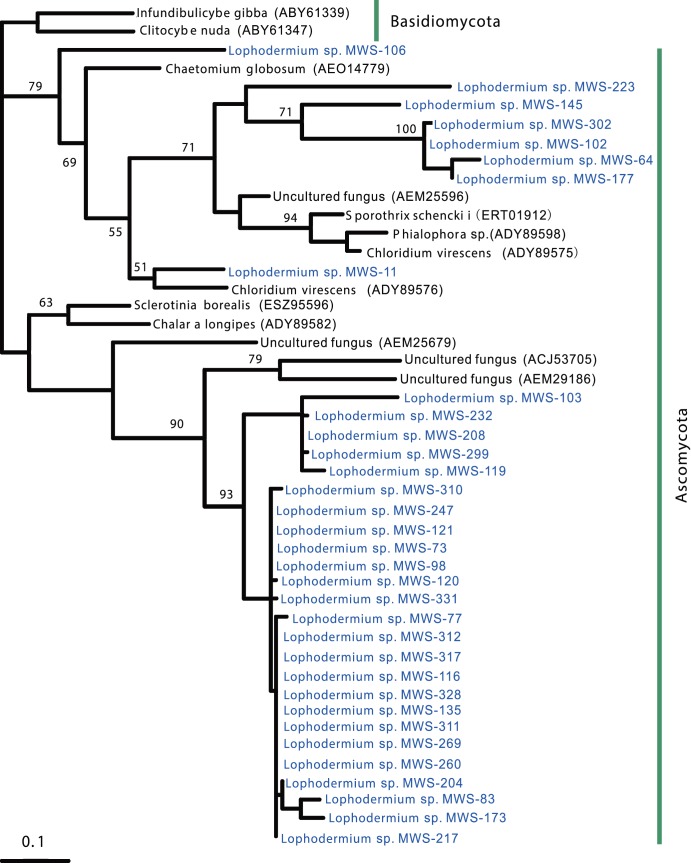
Phylogenetic tree of the partial cellobiohydrolase amino-acid sequences of the *Lophodermium* isolates and their close relatives. Bootstrap values greater than 50% are indicated at branch nodes.

**Figure 7:**
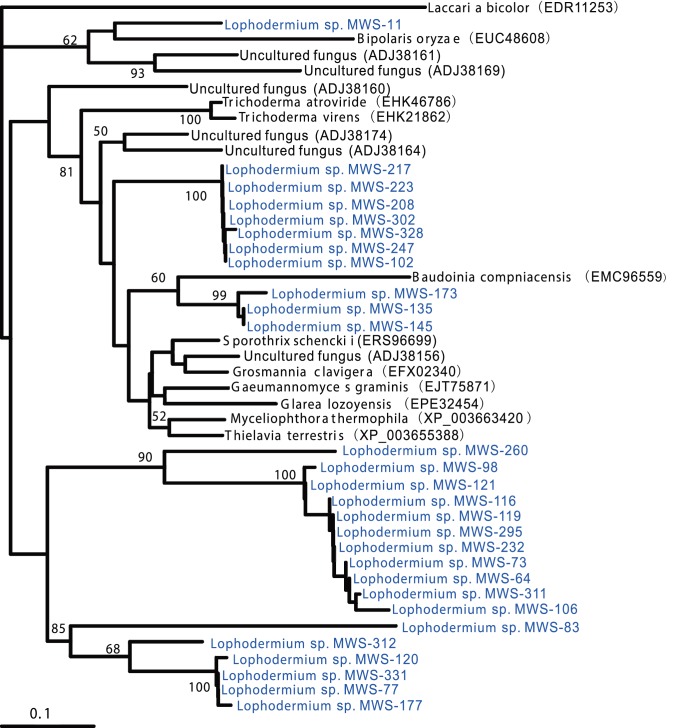
Phylogenetic tree of the partial β-glucosidase amino-acid sequences of the *Lophodermium* isolates and their close relatives. Bootstrap values greater than 50% are indicated at branch nodes.

**Figure 8:**
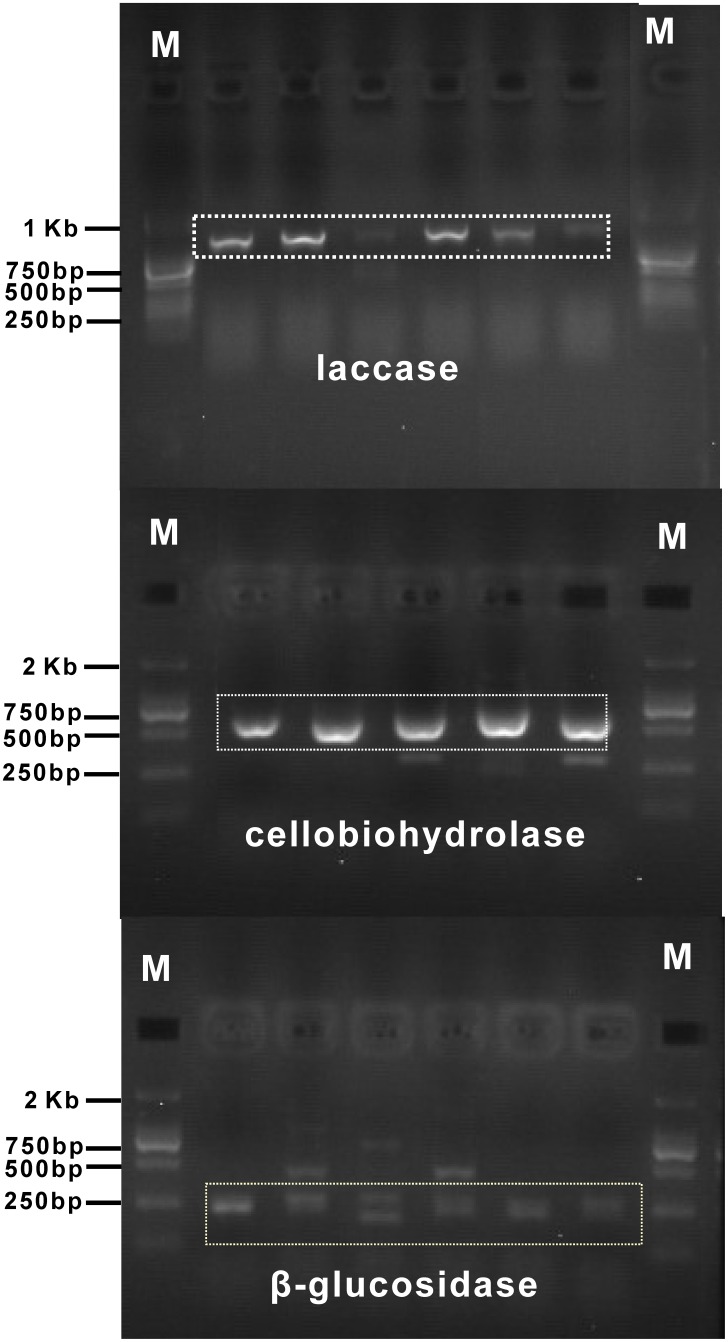
Gel electrophoresis of the amplified partial genes of β-glucosidase, laccase and cellobiohydrolase from *Lophodermium* isolates. “M” indicates the size marker.

Laccase, cellobiohydrolase, and β-glucosidase genes were identified in endophytes (Figs. 5, 6 and 7). Specific degenerate primer pairs were used to amplify the corresponding gene fragments. Agarose gel electrophoresis revealed PCR products of the expected size (approximately 250, 950 and 500 bp for β-glucosidase, laccase and cellobiohydrolase, respectively; Fig. 8). After the introns were removed, the average lengths of the gene fragments were 230, 890 and 430 bp, respectively. The laccase and cellobiohydrolase genes were easily amplified compared to the β-glucosidase encoding gene, for which only faint bands were generated. Most *Lophodermium* isolates yielded positive results, but a very small proportion generated negative results despite the optimization of PCR conditions (Table S1), indicating either the absence of related genes or sequence divergence in the primer-binding regions. Searches with BLASTx and BLASTp confirmed that these sequences corresponded to the three functional enzymes.

In contrast to the phylogeny deduced from the ITS sequencing, the three genes from *Lophodermium* isolates suggest diversity within this genus. The phylogenies inferred from the partial amino acid sequences (AAS) of β-glucosidase, laccase and cellobiohydrolase in endophytic *Lophodermium* isolates diverged from those of other fungi, and at least 20, 20 and 25 distinct sequences were detected for these enzymes, respectively. All AAS showed low similarities to known ascomycete enzymes, with 42–65% identity. We did not retrieve any AAS for *Lophodermium* from the database. This finding further supported the hypothesis that *Lophodermium* isolates form unique phylogenetic branches. Interestingly, the phylogenies also demonstrated that some isolates differed far more than would have been expected based on the near identity of their ITS sequences (for example, isolates MWS-173, MWS-116, and MWS-103).

## Discussion

There is strong evidence that the rate of decomposition is higher in deciduous than in coniferous forests (because of both litter quality and soil acidity). Needle decomposition is largely carried out by fungi due to their ability to degrade recalcitrant polymers such as lignocellulose complexes [10]. There is a large body of literature investigating microbial succession in needle litter [5], revealing the synergistic action of ligninolytic and cellulolytic fungi [10], [30].

Recent studies of the composition of litter-associated fungi revealed that FEs are frequently detected in litter [13]. The exoenzymes produced by endophytes also suggested that FEs are potentially important decomposers [18], [25], [31]; however, the extent to which FEs contribute to litter loss is less well known and somewhat controversial. In this study, we adopted both *in vitro* and *in vivo* approaches to assess the contributions of individual FEs and natural FE populations to needle decomposition. Our data support the hypothesis that FE saprophytism and endophytism may be a prerequisite for rapid decomposition and thus for FEs’ contribution to litter decomposition.

*Lophodermium* spp. are the dominant endophytic colonizers in spruce and pine, and their role in litter decomposition has recently become more clear. Müller *et al*. [9] first reported that *L. piceae*, the dominant fungus of Norway spruce (*Picea abies*), was not an important decomposer, as it was infrequently found after several months of incubation. This conclusion, however, was not supported by Korkama-Rajala *et al*. [3], who suggested that relatively high temperatures inhibit the sporulation of *L. piceae*. The latter researchers developed an rRNA-based denaturing gradient-gel electrophoresis (DGGE) method and found that *L. piceae* remained metabolically active even after two years of decay. *L. pinastri*, a dominant facultative endophyte in *Pinus sylvestris*, was commonly detected in needle litter [24]. An *in vitro* test also showed that *L. pinastri* was capable of decomposing cellulose and lignin.

In the present work, we did not aim to track the dynamics of *Lophodermium* spp. over different incubation times. Our focus was on the genetic and enzymatic basis of decomposition initiation by *Lophodermium* spp. To the best of our knowledge, this is the first report addressing the occurrence of β-glucosidase, laccase and cellobiohydrolase genes in *Lophodermium*. The relatively high divergence of amino acid sequences suggests a flexible and delicate decomposition system involving large numbers of *Lophodermium* and conferring an ecological advantage in competition for nutrients [21]. Šnajdr *et al*. [10] showed that β-glucosidase and cellobiohydrolase were essential for early litter decomposition, and in later stages, laccase and other ligninolytic enzymes largely determine the decomposition rate. The co-occurrence of these three genes in *Lophodermium* isolates reinforces the hypothesis proposed by Korkama-Rajala *et al*. [3] and provides direct evidence for the involvement of *Lophodermium* in decomposition. We selected these three genes as functional markers because of their importance in lignocellulose degradation and ease of amplification from genomic DNA. Kellner and Vandenbol [8] introduced a series of primer pairs to amplify many ligninolytic enzyme-related genes, facilitating a fuller understanding of the ecology of fungi in litter. Little is known about the genetic basis of ligninolytic enzyme activity in FEs or pure cultures. An important next step will be the construction of metagenomic libraries of litter-associated FEs, enabling a robust analysis of multiple functional markers.

Our results demonstrate that there is no significant difference between the two *Lophodermium* taxa in terms of decomposition ability, but it remains unknown how long these taxa can survive in the litter and how they compare in terms of survival. Very few studies have found more than one endophytic *Lophodermium* species in a single coniferous host. The design of multi-taxon-specific probes would enable a comparison of the persistence and activity between the two taxa.

From the *in vivo* experiment, we found that a linear decay model was the best fit for FE-triggered needle decomposition, implying that the FE community may stably facilitate decomposition. Although the incubation temperature and relative humidity most likely differ from climatic conditions, the loss of approximately 60% of mass after seven months of incubation strongly suggests that natural FE communities actively participate in litter decomposition in the early stage. This result is perfectly consistent with the conclusions of other recent studies [5], [24]. One weakness of this work is that the contribution of non-endophytes and that of endophytes plus non-endophytes were not considered. It is relatively easy to evaluate the contribution of whole microbial groups (epiphytes and endophytes) by using intact needles without surface sterilization for incubation, but it is difficult to eliminate endophytes while sustaining epiphytes. One potential approach would be for the autoclaved needles to be incubated on non-sterilized soils.

**Figure 9:**
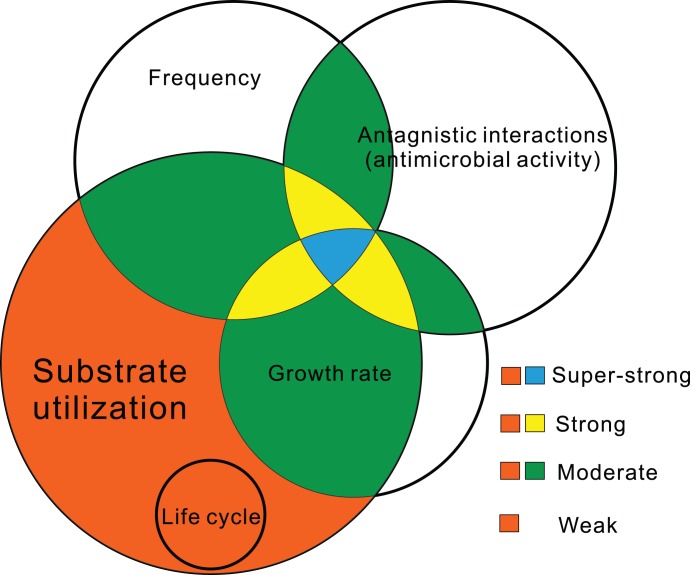
Potential indicators for assessing FE-mediated decomposition. A wide range of substrate-utilization patterns (ligninolytic enzymes) serves as a prerequisite for decomposition. The high colonization frequency reflects the large number of niches occupied by FEs. Antibiotic metabolites produced by FEs would confer a major ecological advantage in competition with other microbial groups. A rapid growth rate guarantees the persistence of mycelium on substrates (litter) for nutrition acquisition. Life cycles differ greatly among fungi. The role of FE life cycles in decomposition is not well known. Most often, FEs are sterile on artificial media, and their life cycles in litter are not well characterized.

Relying on molecular techniques, studies of succession in fungal communities in decomposing litter usually conclude that the detection frequency of certain fungal members is correlated with their decomposition ability; however, this is not always the case [31]. Life cycle, substrate utilization and antagonistic interactions more accurately reflect the performance of FEs in litter [14]. We have previously shown that Dermateaceae spp. and *Pezicula sporulosa* (now identified as a new species, *P. neosporulosa*) [34] dominate on *Abies beshanzuensis* needles and are strong decomposers. Their activity is due not only to their production of laccase but also their moderate or strong anti-fungal activity [21]. The two *Lophodermium* taxa showed no antifungal activity in this study, but *Lophodermium* spp. grow more quickly than Dermateaceae spp. and *P. neosporulosa* on artificial media. Similarly, a fast-growing endophytic Schizophyllaceae sp. (Agaricomycetes) in *A. beshanzuensis* causes the highest mass loss in that community despite its very low frequency and lack of antimicrobial activity [21]. This result suggests that the fungal growth rate may constitute an additional indicator of decomposition activity. We present here a summary of potential indicators by which to assess endophyte-mediated decomposition and identify the core species in the community (Fig. 9). We recommend a polyphasic approach, including molecular ecology, phylogeny, basic biology, and physiology, to fully understand the role of FEs in litter decomposition.

## Supporting Information

Table S1 Detection of β-glucosidase, laccase and cellobiohydrolase gene expression in endophytic *Lophodermium* taxa, and dry weight loss of autoclaved needles elicited by selected isolates *in vitro*.
(DOCX)
