# Supplementary material for: The Role of Endophytic Fungal Individuals and Communities in the Decomposition of Pinus massoniana Needle Litter
Source: PLoS One. 2014 Aug 26;9(8):e105911. doi: 10.1371/journal.pone.0105911 (PMC4144953; doi:10.1371/journal.pone.0105911)
Supplement: Table S1 — Detection of β-glucosidase, laccase and cellobiohydrolase gene expression in endophytic Lophodermium taxa, and dry weight loss of autoclaved needles elicited by selected isolates in vitro. (DOCX) [file pone.0105911.s001.docx]

**Table S1.** Detection of β-glucosidase, laccase and cellobiohydrolase gene expression in endophytic *Lophodermium* taxa, and dry weight loss of autoclaved needles elicited by selected isolates *in vitro*. ND=not determined. “+” indicated positive PCR reaction,” **─**” indicated negative PCR reaction.

|  | Isolates | PCR products | | | Mass loss (%) |
| --- | --- | --- | --- | --- | --- |
|  |  | β-glucosidase | laccase | cellobiohydrolase |  |
| *Lophodermium* sp. 1 | MWS-269 | **─** | + | + | 16.35 ± 1.23 |
|  | MWS-208 | + | + | + | ND |
|  | MWS-73 | + | **─** | + | ND |
|  | MWS-302 | + | + | + | 17.95 ± 1.80 |
|  | MWS-77 | + | **─** | + | ND |
|  | MWS-103 | **─** | + | + | ND |
|  | MWS-331 | + | + | + | ND |
|  | MWS-223 | + | **─** | + | ND |
|  | MWS-106 | + | + | + | ND |
|  | MWS-177 | + | + | + | ND |
|  | MWS-173 | + | **─** | + | ND |
|  | MWS-11 | + | **─** | + | 15.91 ± 0.41 |
|  | MWS-64 | + | + | + | 22.17 ± 0.60 |
|  | MWS-119 | + | + | + | ND |
|  | MWS-232 | + | **─** | + | ND |
|  | MWS-295 | + | + | **─** | 15.37 ± 3.40 |
|  | MWS-135 | + | + | + | ND |
|  | MWS-116 | + | + | + | 14.66 ± 0.40 |
|  | MWS-299 | **─** | + | + | 17.52 ± 2.85 |
|  | MWS-120 | + | + | + | ND |
| *Lophodermium* sp. 2 | MWS-204 | **─** | + | + | 22.34 ± 4.2 |
|  | MWS-328 | + | + | + | 18.32 ± 6.2 |
|  | MWS-312 | + | + | + | ND |
|  | MWS-311 | + | + | + | ND |
|  | MWS-260 | + | + | + | ND |
|  | MWS-83 | + | + | + | ND |
|  | MWS-98 | + | + | + | ND |
|  | MWS-121 | + | + | + | ND |
|  | MWS-102 | + | + | + | ND |
|  | MWS-317 | **─** | + | + | 18.07 ± 2.50 |
|  | MWS-217 | + | + | + | 15.09 ± 1.90 |
|  | MWS-145 | + | + | + | ND |
|  | MWS-310 | **─** | + | + | 24.96 ± 1.30 |
|  | MWS-247 | + | + | + | 16.50 ± 1.60 |
